# Development of an Ion-Pairing Reagent and HPLC-UV Method for the Detection and Quantification of Six Water-Soluble Vitamins in Animal Feed

**DOI:** 10.1155/2016/8357358

**Published:** 2016-07-31

**Authors:** Ho Jin Kim

**Affiliations:** National Agricultural Products Quality Management Service, Gimcheon 740-871, Republic of Korea

## Abstract

A novel and simple method for detecting six water-soluble vitamins in animal feed using high performance liquid chromatography equipped with a photodiode array detector (HPLC/PDA) and ion-pairing reagent was developed. The chromatographic peaks of the six water-soluble vitamins were successfully identified by comparing their retention times and UV spectra with reference standards. The mobile phase was composed of buffers A (5 mM PICB-6 in 0.1% CH_3_COOH) and B (5 mM PICB-6 in 65% methanol). All peaks were detected using a wavelength of 270 nm. Method validation was performed in terms of linearity, sensitivity, selectivity, accuracy, and precision. The limits of detection (LODs) for the instrument employed in these experiments ranged from 25 to 197 *μ*g/kg, and the limits of quantification (LOQs) ranged from 84 to 658 *μ*g/kg. Average recoveries of the six water-soluble vitamins ranged from 82.3% to 98.9%. Method replication resulted in intraday and interday peak area variation of <5.6%. The developed method was specific and reliable and is therefore suitable for the routine analysis of water-soluble vitamins in animal feed.

## 1. Introduction

Vitamins are essential for nutrition and physiological function, and therefore, their intake is necessary for humans and animals, as the body is unable to synthesize these essential nutrients required for growth [1]. Vitamins can be classified into water-soluble and fat-soluble vitamins; the former includes niacin, nicotinamide, vitamin B6, vitamin B2, vitamin B1, folic acid, pantothenic acid, and vitamin C, while the latter includes vitamin A, vitamin D, vitamin E, and vitamin K [2]. Vitamins have specific and important functions, and as such, both humans and animals require vitamins to achieve and maintain health and productivity [3]. Vitamins function to control various endogenous metabolic activities taking place in the body, such as energy and amino acid metabolism [4]. Although only small amounts of vitamins are required, vitamin deficiency (or indeed, excess) can lead to diseases such as beriberi [5] and dermatitis [2, 6, 7]. Thus, to prevent a vitamin deficiency or excess in animals, as well as to attain maximum performance, the control of feed additives is necessary [8]. Indeed, vitamins are essential for maintaining normal metabolic processes in animals, while also retaining their condition and performance. As animals are unable to synthesize vitamins, the small amounts of these nutrients required must be supplied in their feed [9].

Various methods have been reported for the characterization of water-soluble vitamins, such as high performance liquid chromatography (HPLC) coupled with ultraviolet (UV) detection [10, 11], liquid chromatography (LC) coupled with mass spectrometry (MS) [12], LC coupled with MS/MS [13], and gas chromatography [14]. The most commonly used methods for the determination of vitamin components are based on HPLC separation. LC-MS and LC-MS/MS, which provide information about the molecular mass and structural features of components, are considered more useful than other methods for the separation, identification, and quantification of the characteristic vitamin compounds. However, since these methods are expensive to purchase and maintain, many laboratories prefer HPLC-UV detection [15], which is less costly, relatively convenient to operate, and suitable for the routine analysis of vitamins. Ion-paring reagent we used in this study has amine group, so it is suitable for water-soluble vitamins analysis as well as iodine speciation analysis and pesticide residue analysis [16–18].

**Figure 1:**
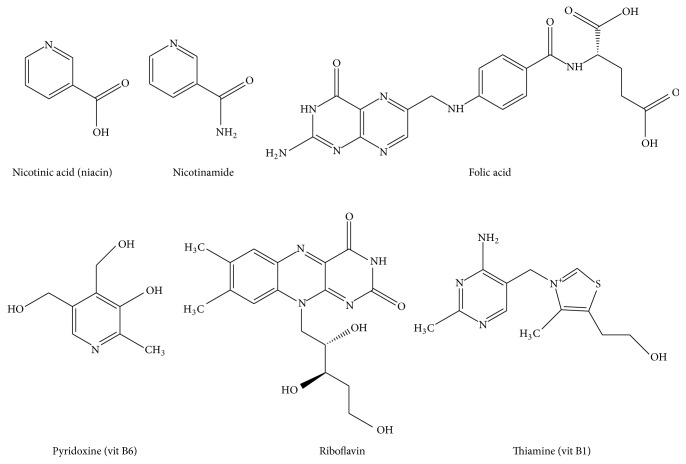
Chemical structures of six water-soluble vitamins analyzed in this study.

In this study, six water-soluble vitamins in animal feed (nicotinic acid (niacin), nicotinamide, folic acid, riboflavin, pyridoxine (vitamin B6), and thiamine (vitamin B1)) (Figure 1) were analyzed simultaneously. Complete animal feeds have complex matrixes containing fat, protein, carbohydrate, salt, and so on. Water-soluble vitamins we targeted have amine functional group which represents polarity. To increase the selectivity of the water-soluble vitamins in complicated matrixes, these nontarget matrixes should be removed. In addition, simultaneous analytical method for the determination of 6 water-soluble vitamins has been rarely reported. Moreover, AOAC method or other international methods each have vitamin method. Therefore, this newly developed simultaneous method should be validated in various feed matrixes. A novel analytic method is therefore reported, which is suitable for detecting and quantifying the vitamins present in animal feed, while also aiding in the management of feed standards.

## 2. Experimental

### 2.1. Samples

The water-soluble vitamins present in animal feed were targeted in this study. Animal feed samples were purchased from a local supermarket. A total of 30 animal feed samples were used, and 20 dried samples were pulverized into fine powders (HMF-100, HANIL Electric Co., Seoul, Korea). The pulverizer was set at a maximum speed of 22,000 rpm to give fine powders ranging in size from 400 to 1000 *μ*m. The remaining 10 liquefied samples were used as received. All samples were stored at 4°C.

### 2.2. Chemicals and Reagents

HPLC grade acetonitrile, methanol, and acetic acid were purchased from Merck (Darmstadt, Germany), and PICB-6 (PIC, paired-ion chromatography; B, separate bases) was purchased from Waters (St. Milford, MA, USA). Water was purified using a Milli-Q® RiOs*™*/Elix® water purification system (Millipore, Bedford, MA, USA). Nicotinic acid (niacin), nicotinamide, pyridoxine (vitamin B6), riboflavin, thiamine (vitamin B1), and folic acid standards were purchased from Sigma Aldrich Chemie GmbH (Bellefonte, PA, USA). All other chemicals and solvents were of reagent grade or higher.

### 2.3. Standard Preparation

The six vitamins were classified into three groups, groups 1, 2, and 3, and stock solutions were prepared using the preprocessing reagent, water, and 0.01 N NaOH to obtain a final vitamin concentration of 100 mg/L. Group 1 consisted of thiamine and pyridoxine, and the stock solutions were prepared by mixing the standard preparation with water (100 mL). Group 2 consisted of riboflavin and folic acid and was prepared by mixing the standard preparation (10 mg), 0.01 N NaOH, and water (100 mL). Group 3 consisted of nicotinic acid and nicotinamide and was prepared by mixing the standard preparation (10 mg) with the preprocessing reagent (100 mL). Working solutions were prepared by diluting the stock solutions, and all solutions were refrigerated.

### 2.4. Sample Preparation

The samples were prepared for extraction by the ion-pairing reagent. Each homogenized sample from animal feed (1.5 g) was placed into a centrifuge tube and mixed with the extraction solvent (10 mL, 5 mM PICB-6 in 0.1% CH_3_COOH). A portion of the liquefied sample (10 g) was added to a 25 mL volumetric flask, and the volume was made up to 25 mL using the extraction solvent (5 mM PICB-6 in 0.1% CH_3_COOH). We used PICB-6 as ion-pairing reagent because most of vitamins we determined showed higher resolution when we used PICB-6 than other ion-pairing reagents. The mixtures were then homogenized for 10 min, sonicated for 10 min at room temperature, and centrifuged at 13,000 rpm for 10 min at 4°C. The resulting supernatant was filtered through a 0.45 *μ*m disposable filter (Whatman). All measurements were performed in triplicate. Finally, the stabilities of the standard preparations were determined over one week in an amber vial at room temperature, with samples being taken for analysis every 24 h.

### 2.5. HPLC Analysis

HPLC was performed on a Shiseido Nanospace SI-2 system (Shiseido, Tokyo, Japan) equipped with a binary solvent delivery pump, an autosampler, and a photodiode array detector (PDA) and was controlled using the EZChrome Elite software (Agilent Technologies, Palo Alto, CA, USA). A reversed phase Unison UK-C18 (100 m × 4.6 mm, 3 *μ*m particle size) (Tokyo, Japan) column was used for all separations at a column temperature of 40°C. Prior to use, the mobile phase was filtered through a 0.45 *μ*m membrane filter (Millipore, Milford, MA, USA) and degassed under vacuum. The mobile phase was composed of buffers A (5 mM PICB-6 in 0.1% CH_3_COOH) and B (5 mM PICB-6 in 65% methanol) with the following gradient elution: 0 min, 10% B; 0–2 min, 10% B; 2–22 min, 70% B; 22–27 min, 70% B; 27-28 min, 10% B; and 28–35 min, 10% B. The sample injection volume was 2 *μ*L, and the flow rate was set at 0.5 mL/min. All peaks were detected using a wavelength of 270 nm.

### 2.6. Method Validation

**Figure 2:**
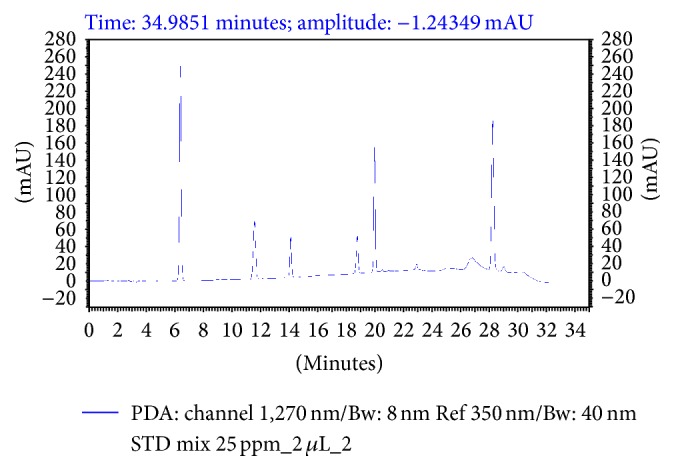
HPLC chromatograms of a mixture of six vitamins detected at 270 nm. (1) Nicotinic acid, (2) nicotinamide, (3) pyridoxine, (4) folic acid, (5) riboflavin, and (6) thiamin.

Method validation was performed according to the guidelines set by the International Conference on Harmonization (ICH, 2005) [18] and the International Union of Pure and Applied Chemistry (IUPAC, 2002) [19]. The method was validated for linearity, sensitivity, selectivity, accuracy, and precision, as outlined in Figure 2 and Table 1.

## 3. Results and Discussion

### 3.1. Method Validation

The developed RP-HPLC/UV method incorporating an ion-pairing reagent was validated to verify that its performance met the requirements for routine vitamin analysis. Several performance characteristics were measured, including selectivity, linearity, sensitivity, accuracy, and precision.

Selectivity was determined by the absence of interference in the chromatographic window, and this was measured using blank chromatograms at the specific quantification wavelength (i.e., 270 nm). As shown in Figure 2 and Table 1, the chromatograms of the water-soluble vitamins indicate successful separation of all six compounds in <29 min, with good resolution (5.55–34.85) and asymmetry (0.94–1.08), thereby indicating satisfactory selectivity for this HPLC system.

Linearity was assessed by building external calibration curves for each compound using the vitamin-containing working solutions. Calibration curves were obtained by plotting the analyte peak area versus its concentration over seven different concentrations. Each concentration of the mixed standard solution was injected in triplicate, and then, the regression parameters were calculated. The results are shown in Table 1. Correlation coefficients (*r* ^2^ > 0.999) were obtained for all compounds studied. These results demonstrate that an external standard calibration can be applied for quantitative purposes.

The sensitivity of the developed method was evaluated by determining the LOD and LOQ values. Under the described chromatographic conditions, these values were calculated based on the response and slope of each regression equation at signal-to-noise ratios (*S*/*N*) of 3 : 1 and 10 : 1, respectively. For the different components, the LOD values ranged from 25 to 197 *μ*g/kg, while the LOQ values ranged from 84 to 658 *μ*g/kg. Detailed data are shown in Table 1.

The method precision was then determined by measuring the intra- and interday precision. For the intraday precision, six replicates of the mixed standard solutions were analyzed within 1 d, while the solutions were examined in triplicate for three consecutive days for the intraday precision. The precision was expressed as the percentage of the relative standard deviation (% RSD). The overall intraday % RSD values were <5.6%, while the interday values were <4.8% (see Table 1).

The accuracy was evaluated by adding the mixed standard solutions at two different concentrations (high: 20.0 mg/kg; low: 2.0 mg/kg) to feed from the Association of American Feed Control Official (AFFCO, 201591-Swine Mineral and Vitamin Supplement). The mixture was extracted using the developed ion-pairing reagent technique and HPLC method. All tests were performed in triplicate. Excellent recovery rates of 82.3–98.9% indicated a high level of accuracy for this method, as detailed in Table 1.

The stability of all standard solutions was also tested over 7 d, with the majority having decomposed within 3 d. In particular, in the case of folic acid, rapid decomposition was observed after 3 d compared to that of the other water-soluble vitamins (i.e., 63.38% cf. 92.12–95.93%). These results demonstrated that it is necessary to prepare a fresh standard preparation for each analysis. Detailed results are shown in Table 2.

Based on the above validation data, the proposed method was concluded to provide good linearity, sensitivity, selectivity, accuracy, and precision for the simultaneous analysis of water-soluble vitamins.

### 3.2. Application of the Developed Method

Among the 30 different animal feeds that were analyzed in this study, vitamins were detected in all animal feeds purchased from supermarkets (Table 3). Each sample was analyzed in triplicate. Identification of the six compounds was by comparison of their retention times and UV spectra with those of the standards and the pure compounds. The qualitative and quantitative compositions of the six compounds in the animal feed varied significantly. More specifically, the nicotinic acid content ranged from 1,868 to 46,289 mg/kg with an average content of 15,969 mg/kg, while the nicotinamide content ranged from 1,339 to 49,920 mg/kg with an average of 16,257 mg/kg. In addition, the content of folic acid varied between 102 and 5,012 mg/kg (average = 813 mg/kg), while that of pyridoxine was between 2,065 and 49,530 mg/kg (average = 17,758 mg/kg). Finally, the riboflavin and thiamine contents ranged from 3,015 to 36,820 mg/kg (average = 13,286 mg/kg) and from 1,359 to 48,930 mg/kg (average = 11,786 mg/kg), respectively.

Thus, a simple, qualitative, and quantitative method for the simultaneous detection and quantification of six vitamin compounds from animal feed was successfully developed and validated using RP-HPLC/UV detection. Furthermore, the extraction process was optimized using an ion-pairing reagent. The proposed method showed accuracy and precision and was successfully employed for analyzing different types of feeds. Analytical results demonstrated that this HPLC method provides a good alternative for routine analysis, owing to its simplicity, specificity, and sensitivity. Finally, it exhibits the potential to be applied as a reliable quality evaluation method for animal feed.

## Figures and Tables

**Table 1:** Validation parameters for the developed RP-HPLC/UV method.

Compound	RT^a^	Slope	Intercept	*r* ^2^ ^b^	LOD^c^ (*μ*g/kg)	LOQ^d^ (*μ*g/kg)	Rs^e^	Asymm^f^	Precisions (% RSD^g^)		Recovery^h^ (%)
									Intraday (*n* = 6)	Interday (*n* = 9)	
Nicotinic acid	6.4	1.3365	−7.2254 × 10^−1^	0.9999	31	105	—	1.07	2.6	3.0	94.5
Nicotinamide	11.6	2.3354	−5.4255 × 10^−1^	0.9999	25	84	19.42	0.97	2.2	2.1	93.5
Pyridoxine	14.2	8.5554 × 10^−1^	−2.33664	0.9998	197	658	10.20	0.99	2.2	3.0	90.6
Folic acid	18.8	1.22248	1.00014 × 10^−1^	0.9997	104	347	21.49	0.99	3.0	3.3	82.3
Riboflavin	20.1	2.65487	−5.66541 × 10^−1^	0.9999	33	110	5.55	0.94	5.6	4.8	95.2
Thiamin	28.3	3.26548 × 10^−1^	−1.02548 × 10^−1^	0.9997	28	94	34.85	1.08	4.0	4.2	98.9

^a^Retention time (min).

^b^Coefficients of correlation.

^c^Limit of detection, the lowest analyte concentration that produces a response detectable above the noise level of the system.

^d^Limit of quantification, the lowest level of analyte that can be accurately and precisely measured.

^e^Resolution.

^f^Asymmetry.

^g^Relative standard, expressed as %.

^h^Average of recoveries at two spike levels (high and low).

**Table 2:** Stability of six water-soluble vitamins.

Storage time (d)	Nicotinic acid	Nicotinamide	Folic acid	Pyridoxine	Riboflavin	Thiamine
1	98.70 ± 0.05	97.54 ± 0.14	99.83 ± 0.21	98.58 ± 0.02	97.66 ± 1.10	99.24 ± 0.20
2	96.57 ± 0.03	96.03 ± 0.02	89.01 ± 0.02	96.12 ± 0.54	95.69 ± 0.52	97.10 ± 0.66
3	94.29 ± 0.12	92.12 ± 0.24	63.38 ± 0.05	93.03 ± 0.86	94.44 ± 0.99	95.93 ± 1.65
4	85.32 ± 0.08	83.66 ± 1.03	60.93 ± 1.21	84.42 ± 0.39	84.93 ± 1.63	87.18 ± 1.87
5	70.85 ± 0.07	73.25 ± 0.08	56.24 ± 0.34	73.15 ± 1.09	71.03 ± 0.83	72.93 ± 0.39
6	63.03 ± 0.16	67.26 ± 0.12	52.62 ± 0.44	65.05 ± 1.21	66.38 ± 0.41	62.01 ± 0.33
7	59.63 ± 0.13	59.90 ± 1.08	49.59 ± 0.20	60.99 ± 0.50	58.99 ± 0.62	59.56 ± 0.72

**Table 3:** Determination of the vitamin contents (mg/kg) in animal feed using the proposed method.

Sample	Nicotinic acid	Nicotinamide	Folic acid	Pyridoxine	Riboflavin	Thiamine
1	5,231	6,380	—	20,449	—	10,648
2	1,868	42,085	102	—	—	9,803
3	20,009	—	—	9,027	4,093	—
4	46,289	—	452	—	29,344	8,014
5	9,227	8,263	—	10,382	30,298	—
6	19,263	—	780	37,030	—	5,226
7	—	49,920	—	2,009	—	5,814
8	—	2,892	358	—	10,023	20,687
9	16,354	34,893	—	5,934	3,015	—
10	8,308	—	552	—	23,589	—
11	5,360	7,099	837	—	24,263	18,893
12	—	10,298	960	9,982	—	4,300
13	6,993	26,407	200	6,057	10,360	—
14	21,960	48,062	—	2,918	5,430	9,366
15	—	—	753	—	—	48,930
16	—	6,337	200	49,530	—	1,380
17	40,826	12,086	—	—	5,669	4,085
18	3,698	—	901	8,006	7,802	30,569
19	—	1,339	5,012	—	9,829	—
20	—	—	303	39,807	4,369	—
21	30,158	2,580	—	5,980	—	10,230
22	4,803	—	807	—	36,820	5,628
23	10,009	—	650	48,301	—	7,083
24	—	15,369	—	2,065		8,292
25	25,801	—	—	—	6,043	—
26	26,990	—	268	—	8,823	1,359
27	9,045	—	—	4,6933	—	4,860
28	4,960	9,963	—	—	—	—
29	—	2,860	—	10,297	6,099	—
30	18,203	5,802	692	4,950	—	20,560
